# On the value of the ‘subjective’ in studies of human behavior and cognition

**DOI:** 10.1007/s40037-015-0154-3

**Published:** 2015-01-29

**Authors:** Mark Goldszmidt, Saad Chahine, Sayra Cristancho, Chris Watling, Lorelei Lingard

**Affiliations:** Health Sciences Addition, Room 115, Schulich School of Medicine and Dentistry, Western University, London, ON N6A 5C1 Canada

Letter in response to: **What people say ≠ what people do**

We write this letter as a response to the letter ‘What people say ≠ what people do’, in which Dr. van Merriënboer acknowledges the generic value of subjective data but argues that they are unreliable, misleading, and best combined with objective data in the study of behaviour and cognitive processes [1]. As a research group studying teaching and learning in naturalistic clinical settings, we would like to offer a rejoinder.

First, we question the dichotomous characterization of data as *either* subjective *or* objective. We argue that a spectrum, rather than a dichotomy, exists between ‘subjectivity’ and ‘objectivity’ in research data. At one end of the spectrum are the research approaches Van Merriënboer refers to that ‘[ask] people their opinions’ using interviews, while at the other end are research approaches that attempt to purge all external human influence. Between these poles, degrees of ‘subjectivity’ and ‘objectivity’ exist. Van Merriënboer’s focus on interview techniques that seek opinions belies the richness and diversity of naturalistic data collection methods, which can employ photography, critical incident interview techniques and video-recording of human experiences. Such data are not straightforwardly ‘subjective’: they combine, often in nuanced ways, both more subjective (influenced by human interpretation) and more objective (unfiltered representation) dimensions. Furthermore, the most ‘objective’ research – such as eye tracking analyses or double-blind randomized controlled trials – has subjective dimensions: it is necessarily influenced by human interpretation, from the wording of the question asked, to the inclusion/exclusion criteria of the sampling and the selection of statistical tests [2].

Second, we respectfully disagree with Van Merriënboer’s marginalization of ‘subjective data’ in the study of cognition and behaviour. Calls for studying cognition and behaviour outside of the laboratory [3, 4] suggest that naturalistic research generally, and interview techniques in particular [5, 6] can offer meaningful insights into cognition and behaviour. Furthermore, many techniques exist for enhancing the rigour and authenticity of interview data regarding human cognition and behaviour [5, 6]. Interviews may be framed around clinical case presentations to elicit valuable insights into how clinicians work, [7] as in a recent study exploring faculty supervisory practices [8]. The ‘guided walk’ technique enriches interviews with authentic contextual details, as in a recent study of the lived experience of medical students in remote rural communities [9]. And interview protocols that incorporate workplace observations and visual methods can elicit tacit aspects of expert practice [10, 11]. Importantly, these techniques do not reduce subjectivity in the interview. Rather, they enrich interview data with more perspectives, more interpretive resources, more glimpses of the human participants’ implicit and explicit understandings of their work processes.

In conclusion, we suggest putting aside the dichotomy between subjective and objective data. We advocate that medical education researchers draw from the full spectrum of approaches in the exploration of human cognition and behaviour. From our perspective, each methodology and the data it produces have something to contribute; none is intrinsically more valuable.
